# The EZH2 SANT1 domain is a histone reader providing sensitivity to the modification state of the H4 tail

**DOI:** 10.1038/s41598-018-37699-w

**Published:** 2019-01-30

**Authors:** Tyler M. Weaver, Jiachen Liu, Katelyn E. Connelly, Chris Coble, Katayoun Varzavand, Emily C. Dykhuizen, Catherine A. Musselman

**Affiliations:** 1 Department of Biochemistry, Carver College of Medicine, University of Iowa, Iowa City, IA 52242 USA; 2 Department of Medicinal Chemistry and Molecular Pharmacology, College of Pharmacy, Purdue University, West Lafayette, IN 47907 USA

## Abstract

SANT domains are found in a number of chromatin regulators. They contain approximately 50 amino acids and have high similarity to the DNA binding domain of Myb related proteins. Though some SANT domains associate with DNA others have been found to bind unmodified histone tails. There are two SANT domains in Enhancer of Zeste 2 (EZH2), the catalytic subunit of the Polycomb Repressive Complex 2 (PRC2), of unknown function. Here we show that the first SANT domain (SANT1) of EZH2 is a histone binding domain with specificity for the histone H4 N-terminal tail. Using NMR spectroscopy, mutagenesis, and molecular modeling we structurally characterize the SANT1 domain and determine the molecular mechanism of binding to the H4 tail. Though not important for histone binding, we find that the adjacent stimulation response motif (SRM) stabilizes SANT1 and transiently samples its active form in solution. Acetylation of H4K16 (H4K16ac) or acetylation or methylation of H4K20 (H4K20ac and H4K20me3) are seen to abrogate binding of SANT1 to H4, which is consistent with these modifications being anti-correlated with H3K27me3 *in-vivo*. Our results provide significant insight into this important regulatory region of EZH2 and the first characterization of the molecular mechanism of SANT domain histone binding.

## Introduction

Histone proteins are dynamically post-translationally modified during all DNA-templated processes in the eukaryotic cell, and are thought to be one of the main mechanisms by which chromatin structure is regulated in response to cellular signals^1,2^. Histone post-translational modifications (PTMs) have been shown to either alter chromatin structure directly, or recruit or regulate the function of chromatin regulatory proteins and complexes^1^. The latter is mediated through the action of histone binding subdomains known as reader domains, that allow for specific recognition of the histone proteins and their modification state^3–5^. In fact, most chromatin regulators contain multiple histone reader domains, purportedly to enable the read out of complex patterns of histone modification states^6^.

Polycomb Repressive Complex 2 (PRC2) is a histone H3 lysine methyltransferase responsible for generating mono-, di-, and tri-methylation on Lys27 (H3K27me1, H3K27me2 and H3K27me3). The tri-methylated form is known to be critical in gene repression, and its proper placement is essential in defining repression patterns during development^7–9^. Less is known about the biological role of H3K27me2 and H3K27me1, but recently PRC2 dependent H3K27me1 has been linked with active transcription. PRC2 is composed of four main subunits; Suppressor of Zeste 12 (SUZ12), Retinoblastoma-Associated Protein 46/48 (RbAp46/48), Embryonic Ectoderm Development (EED), and the catalytic subunit Enhancer of Zeste Homologue 2 (EZH2). The minimal complex required for a functionally active PRC2 complex contains EZH2, SUZ12 and EED. However, PRC2 activity can be further regulated by a number of accessory subunits, including PHF1, AEBP2, and JARID2^10–14^. In addition, various histone PTMs have been shown to alter PRC2 activity, mediated by histone binding domains in the complex^11,15–18^. For example, the EED WD40 domain has been shown to bind H3K27me3, the catalytic product of PRC2, and allosterically activate the complex^15,19^. The WD40 of RbAp46/48 interacts with unmodified H3K4, providing sensitivity to the H3K4me3 modification associated with active chromatin^17^. In addition to the EED and RbAp46/48 WD40 domains, the EZH2 subunit of PRC2 also contains two SANT domains (named based on their presence in Swi3, Ada2, N-CoR, and TFIIIB). SANT domains contain ~50 amino acids, are structurally homologous to the helix-turn-helix Myb family of DNA binding domains, and are commonly found in chromatin modifying complexes^20^. Though some SANT domains have been shown to interact with DNA, another subset has been found to bind to unmodified histone tails, which is important for regulating the activity of their respective chromatin modifying complexes^21–27^. To date, the functions of the two SANT domains in EZH2 (SANT1 and SANT2) are unknown. The EZH2 SANT domains have little primary sequence homology to classical SANT/Myb domains outside of the core aromatic residues, and in addition SANT1 contains a large insert between predicted helices 1 and 2 (Supplementary Fig. S1a). However, the EZH2 SANT domains are highly conserved across higher eukaryotes from *Drosophila melanogaster* to humans indicating functional importance (Supplementary Fig. S1b). SANT1 and SANT2, on the other hand, share little sequence homology between each other, and have opposite electrostatic properties, suggesting non-redundant function (see Supplementary Fig. S1c).

Recently, several crystal structures of the PRC2 complex were solved^28–30^. The structures suggest that allosteric activation known to occur upon binding H3K27me3 is transmitted through a stimulation response motif (SRM) that is adjacent to SANT1^15,16^. Notably, in the crystal structures containing human EZH2, a large portion of SANT1 had to be deleted in order to facilitate crystallization, thus its fold is not fully understood from these structures^29^. Structures of the basal state (apo EED) compared to the activated state (EED bound to Jarid2K119me3) demonstrate that the SRM becomes structured upon EED association with activating methylated peptide, forming an alpha helix that links the methylated peptide with the catalytic SET domain of EZH2^29,31^. Based on the close proximity of the SRM to SANT1, there has been speculation that SANT1 may be involved in regulating PRC2 activity^28^.

Here we investigate the SRM motif and SANT1 domain of EZH2 in the solution state. We find that the SRM is necessary for stabilization of the adjacent SANT1 domain, and our data suggest that the SRM transiently samples the active helical conformation in solution. In addition, we identify SANT1 as a histone reader domain, with specificity for the unmodified histone H4 N-terminal tail. We define the structural basis of this interaction, which is the first mechanistic insight into any SANT/histone interaction to date. Finally, we show the SANT1 interaction with unmodified H4 is sensitive to the presence of PTMs on H4K16 and H4K20. Together, our results provide valuable insight into this regulatory region of PRC2 and uncover an additional mechanism by which PRC2 can sense the local chromatin landscape via the SANT1 domain.

## Results

### Identification of a minimal stable SANT1 construct

**Figure 1:**
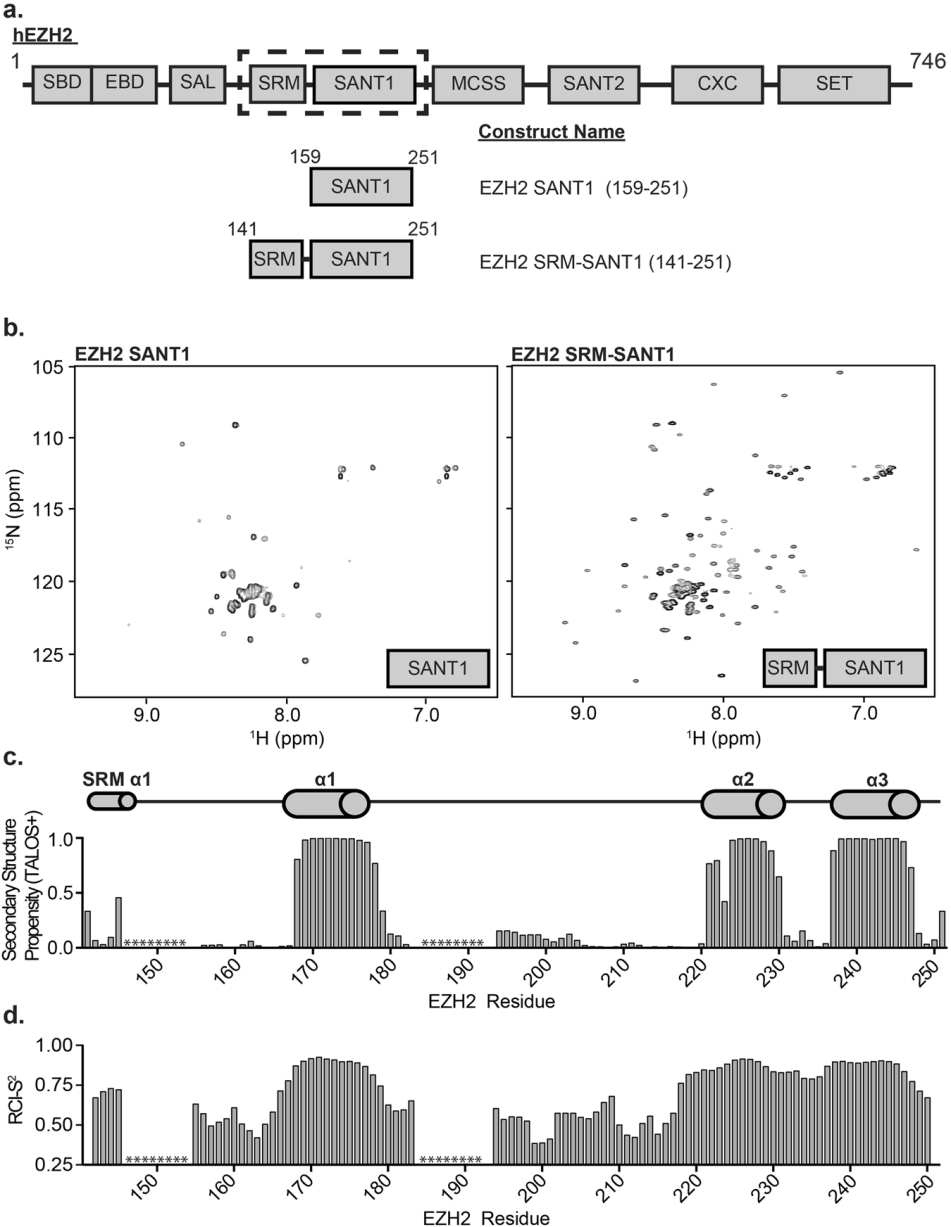
Structural characterization of EZH2 SRM-SANT1. (**a)** The domain architecture of human EZH2. The SRM and SANT1 domain are encased in a dotted black box. The domain architecture of SANT1 and SRM-SANT1 constructs used are shown below. **(b)** An ^1^H-^15^N HSQC spectrum of SANT1 (left) and SRM-SANT1 (right). **(c)** Secondary structure propensity of SRM-SANT1 determined by TALOS+. The predicted secondary structure propensity is shown as a function of residue, ranging between 0 and 1. All predicted secondary structure was alpha helical. * indicates residues with no CO resonance. **(d)** The TALOS+ determined RCI-S^2^ order parameter is shown as a function of residue number where 0 indicates disordered and 1.0 indicates ordered. * indicates residues with no CO resonance.

Though highly conserved, the function of the EZH2 SANT1 domain is currently unknown. In order to investigate this domain, we first identified a minimal stable construct. An initial construct was designed based on domain limit predictions made by the SMART server, which indicate that the minimal structured domain spans residues 159–251 (Fig. 1a)^32^. This initial construct was successfully purified out of *E*. *coli*. However, an ^1^H-^15^N heteronuclear single quantum coherence (HSQC) NMR spectrum revealed only 33 main chain resonances. This is substantially less than what would be expected, based on 88 non-proline residues, and assuming fast conformational exchange on the NMR timescale (Fig. 1b, left). Of the observable resonances, the majority were degenerate in chemical shift lying largely between 7.5ppm and 8.5ppm in the ^1^H dimension. Together this suggests that residues 159–251 are either not sufficient to form a stably folded domain or that the domain is undergoing dramatic conformational dynamics on the intermediate timescale leading to significant line-broadening and disappearance of peaks in the spectrum. In an attempt to stabilize the domain fold, a second construct was generated incorporating an additional 18 residues at the N-terminus, resulting in a construct spanning residues 141–251. This second construct contains a large part of what has now been termed the stimulation response motif (SRM) separated by a small linker (Fig. 1a). An ^1^H-^15^N HSQC spectrum of the SRM-SANT1 construct contained 100 resonances (Fig. 1b, right), revealing that the SRM and SRM-SANT1 linker stabilize the SANT1 domain. The majority of the SRM-SANT1 resonances are well-dispersed in the ^1^H and ^15^N dimensions, suggestive of a well-folded domain. All additional studies were performed with the SRM-SANT1 construct containing residues 141–251.

### Structural Characterization of EZH2 SRM-SANT1

In order to further characterize the SRM-SANT1 construct, backbone amide resonances were assigned through analysis of HNCACB, CBCA(CO)NH, HNCO, HNCACO and HCCH-TOCSY spectra. As shown in Supplementary Fig. S2, 84 resonances were unambiguously assigned. The remaining 16 resonances have largely degenerate chemical shift in both ^1^H and ^15^N and/or correspond to large stretches of degenerate amino acid types precluding assignment. To assess the secondary structure of SRM-SANT1, we analyzed the chemical shifts using Talos+ ^33^. This provides a measure of α-helix or β-sheet propensity on a per residue basis from analysis of the HN, Hα, Cα, Cβ, CO and N chemical shifts. Positive values denote α-helix and negative values β-sheet, with the magnitude indicating the predicted propensity to form these secondary structures. Order parameters (S^2^) can also be predicted, which indicate the magnitude of flexibility (ranging from 0–1, with 1 being the most rigid). Three regions were identified with significant alpha helical propensity corresponding to residues 168–178 (α1 helix), 221–230 (α2 helix) and 237–247 (α3 helix), which corresponds to the SANT domain (Fig. 1c). In addition, a moderate helical propensity is seen for residues in the SRM (Fig. 1c). Correspondingly, a slight increase in predicted order parameters (S^2^) is seen in this region as compared to the random coil regions (Fig. 1d). Together this suggests that the SRM transiently samples a helical conformation. The remaining assigned residues are predicted to be in a random coil conformation. Though the unassigned resonances cannot be fully evaluated the majority of them are degenerate in chemical shift and lie in the middle of the spectrum suggesting that they belong to unstructured regions (Supplementary Fig. S2a).

The predicted secondary structure of the SANT1 domain is consistent with the canonical SANT/Myb domain fold, which is characterized by a three-helix bundle stabilized by the interaction of aromatic residues located in or just outside each of the three helices. Indeed, SANT1 contains three aromatic residues within or just adjacent to each helix; F171 (α1 helix), F224 (α2 helix) and Y244 (α3 helix) (Supplementary Figs S1 and S3). To confirm that these are the stabilizing residues, mutant constructs were generated in which each of these aromatics was substituted with Ala, and scanning wavelength circular dichroism (CD) was used to assess the fold. The CD spectrum of wild-type SANT1 contains negative ellipticity minima at 208 nm and 220 nm, which is consistent with alpha helical secondary structure (Supplementary Fig. S3). In contrast, the CD spectrum of F224A contains a single negative ellipticity minimum at 198 nm, consistent with a completely random coil secondary structure (Supplementary Fig. S3c). The CD spectra of F171A and Y244A have negative ellepticity minima at 208 nm and 220 nm, but show a significant decrease in total ellepticity signal suggesting these mutations significantly destabilize the global structure of the domain without completely unfolding it (Supplementary Fig. S3b,d). Together, this confirms that F171, F224 and Y244 make up the hydrophobic core of SANT1, and that all three residues are required for the domain fold. Notably, a major difference in SANT1 compared to the canonical SANT domains is the size of the α1-α2 loop. Most SANT domains harbor short inter-helical segments comprised of ~5–10 residues (Supplementary Fig. S1). Though the α2-α3 loop is only 7 residues, the α1-α2 loop is extremely long at 42 residues. Our NMR data suggests that this loop is entirely unstructured, which is not surprising considering the low sequence complexity and high percentage of acidic residues (Supplementary Figs S1 and S2). The size of this loop also likely contributes to the increased conformational dynamics observed for the minimal SANT1 construct.

As mentioned previously, during the course of our studies, four crystal structures of PRC2 were solved^28–30^. Minimal complexes of yeast (*Chaetomium thermophilum*), human, and a mixed species (human SUZ12 and EED, and chameleon EZH2) were captured in either the basal, stimulated, or inhibitor-bound states. Importantly residues 183–211, corresponding to the SANT1 α1-α2 loop, were deleted in the complex containing human EZH2 in order to facilitate crystallization^29^. Comparison of our secondary structure analysis to the SANT1 domain in the crystal structure reveals very good agreement, indicating that this deletion did not substantially perturb the SANT1 fold in the crystal structure. The SRM does not have discernable electron density in the basal PRC2 complex lacking activating peptide^30^. Thus it was predicted that the SRM undergoes an unstructured to structured transition upon PRC2 association with peptides corresponding to H3K27me3 (yeast complex) or Jarid2K119me3 (human complex), where it is seen to form an alpha helix^29^. Our results suggest that the SRM transiently samples this active conformation in the isolated state, perhaps contributing to the basal activity of the complex, and suggest that the activating peptide simply stabilizes this conformation upon binding.

### SANT1 is a histone reader domain specific for the H4 tail

**Figure 2:**
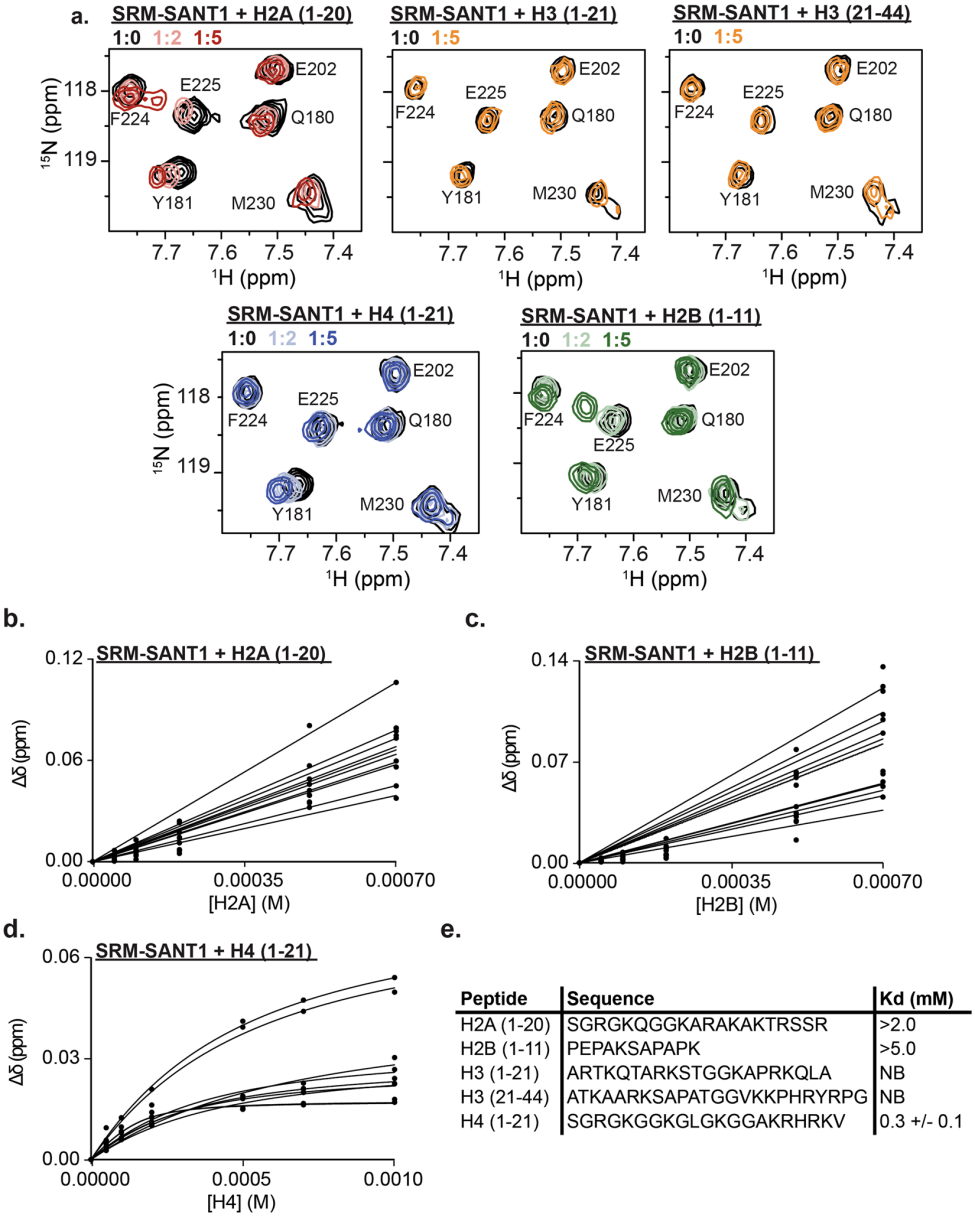
SANT1 is a histone reader domain specific for the H4 tail. (**a)** ^1^H-^15^N HSQC overlay of SRM-SANT1 in the presence of increasing concentrations of H2A (1–20, red), H2B (1–11, green), H3 (1–21, orange), H3 (21–44, orange) and H4 (1–21, blue). Molar ratio of ligand added is indicated in the legend. **(b–d)** All binding curves used to calculate the K_d_ for **(b)** H2A (1–20) peptide, **(c)** H2B (1–11) peptide, or **(d)** H4 (1–21) peptide. **(e)** Table of K_d_s for all histone tail peptides tested. Underdetermined K_d_ values due to lack of saturation are given as lower limits, the H4 value is the average over all significant CSPs and associated standard deviation, NB is no binding.

A subset of SANT/Myb domains has been shown to bind histones, specifically the unmodified histone H3 and H4 N-terminal tails. Recently, several groups have hypothesized that the EZH2 SANT1 domain may be a histone binding SANT domain due to its acidic isoelectric point^30^. To test if EZH2 SANT1 can associate with histone tails, NMR titrations were performed with peptides corresponding to H2A (residues 1–20), H2B (residues 1–11), H3 (residues 1–21), H3 (residues 21–44) and H4 (residues 1–21). Addition of increasing amounts of the unmodified H2A, H2B and H4 peptides resulted in significant CSPs in the SRM-SANT1 spectrum suggesting binding (Fig. 2a and Supplementary Fig. S4). In contrast, addition of increasing amounts of unmodified H3 (1–21 or 21–44), did not lead to any significant CSPs (Fig. 2a and Supplementary Fig. S4). Dissociation constants were computed by fitting the normalized CSPs to a single-site binding model accounting for ligand depletion (Fig. 2b–d). The H4 tail was found to associate with a K_d_ = 0.3+/− 0.1 mM, whereas the H2A and H2B tails bind approximately 10x and 20x weaker, respectively, revealing specificity for the unmodified H4 tail (Fig. 2e).

As classical SANT/Myb domains were originally identified as DNA binding domains, we also investigated the possibility of DNA binding activity. The Myb DBDs have a basic isoelectric point (pI~10), and the interaction with DNA is mediated by the α3 recognition helix. While EZH2 SANT1 has previously been proposed to also bind DNA, it has a highly acidic isoelectric point (pI ~ 4.25). To test the DNA binding, electrophoretic mobility shift assays (EMSAs) and NMR titrations were carried out. Neither analysis showed any association of SRM-SANT1 with DNA (Supplementary Fig. S5).

### Structural Basis for EZH2 SANT1 association with unmodified histone H4

**Figure 3:**
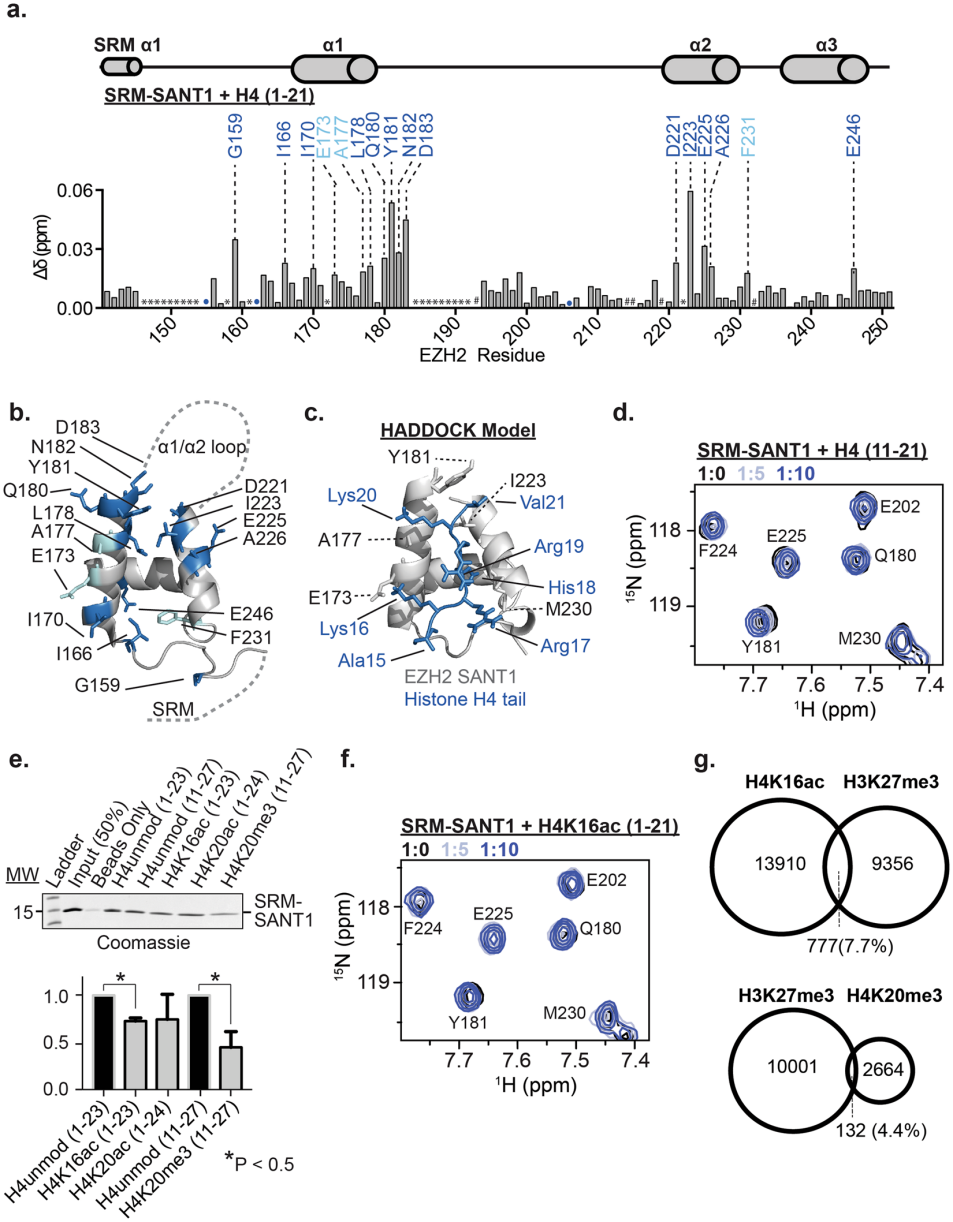
SANT1 provides sensitivity to the modification state of the histone H4 tail. (**a)** Normalized chemical shift perturbation (Δδ) in the ^1^H-^15^N HSQC of ^15^N-SRM-SANT1 between apo and H4-bound as a function of EZH2 residue. TALOS+ predicted secondary structure is shown above. Residues with significant chemical shift perturbation greater than 1.0 and 1.5 standard deviations are labeled with cyan and blue, respectively. Unassigned residues are indicated with *. Prolines are denoted with #. Resonances that broaden beyond detection are labeled with a colored circle. **(b)** Residues with significant CSPs are highlighted as sticks on a cartoon representation of SANT1 (coordinates taken from 5HYN, the α1-α2 loop and SRM are shown as dashed lines). Residues with significant CSPs are colored as in (**a**). **(c)** A HADDOCK model of the SANT1-H4 tail interaction. The histone H4 tail is colored blue and shown as sticks, and the SANT1 domain is shown as a grey cartoon, with interacting residues shown as sticks. SANT1 amino acids are labeled in black using the one-letter code and histone residues are labeled in blue using the three-letter code. **(d)** ^1^H-^15^N HSQC overlay of SRM-SANT1 in the presence of increasing concentrations of H4(11–21) confirms binding to the basic patch of H4 **(e)** Representative biotin-tagged histone peptide pulldown experiments using purified SRM-SANT1 detected by coomassie staining (top, see Supplementary Fig. S9 for full gel). Unmodified or singly modified histone peptides were tested as denoted above the blot. Quantification of three peptide pulldown experiments (bottom). Binding for each modified peptide is compared to the corresponding unmodified control histone peptide. *Denotes significant differences between modified peptides as determined by p-value < 0.05. **(f)** ^1^H-^15^N HSQC overlay of SRM-SANT1 in the presence of increasing concentrations of H4K16ac(1–21) confirms that acetylation of K16 abrogates binding. (**g)** Venn diagrams representing the overlap of H3K27me3 with H4 modifications determined from ChIP-Seq data from IMR90 lung fibroblasts. Shown is the comparison of H3K27me3 with H4K16ac (top) or H4K20me3 (bottom).

In order to identify the molecular basis of interaction with H4, we assessed the normalized change in chemical shift (Δδ) between apo and H4 bound for resonances in the ^1^H-^15^N HSQC spectrum of SRM-SANT1. CSPs report on residues that are directly or indirectly involved in binding. A Δδ was considered significant if it was greater than the average Δδ plus one standard deviation (Fig. 3a). Residues significantly perturbed upon titration of the unmodified H4 tail are located almost exclusively in and around the α1 and α2 helices of SANT1. Mapping these perturbations onto the SANT1 taken from the human PRC2 structure (PDB:5HYN) reveals that these residues form a narrow groove between the α1 and α2 helices consisting of largely hydrophobic residues, with the outside of the binding pocket lined with several acidic residues (Fig. 3b). There were no significant CSPs observed in the SRM, and only one residue perturbed in the SRM-SANT1 linker. Notably, similar analysis for H2A and H2B demonstrates that these peptides lead to broad CSPs across most of SANT1, including in the acidic α1/α2 loop (Supplementary Fig. S6). Combined with the weaker affinities for H2A and H2B, this suggests largely non-specific contacts. The lack of similar non-specific binding with H3, suggests that the enrichment of G/A residues seen in H2A and H2B is important in forming such interactions, likely for purposes of peptide flexibility.

In order to better understand the SANT1/H4 interaction, a model was generated using the program HADDOCK, a data driven docking program^34^. Coordinates for SANT1 were taken from the PRC2 structure (PDB 5HYN). Coordinates for the H4 tail were taken from a nucleosome structure (PDB:1KX5). Restraints were defined based on the NMR CSPs observed for the H4 tail. Specifically, SANT1 residues in α1 (V172, E173, A177, Q180 and Y181) and α2 (D221, K222, I223, E225, A226, M230, T236 and E242) were defined as active residues. Modeling was attempted on the N-terminal portion of the H4 tail (residues 1–15) and the C-terminal region containing the basic patch (residues 15–21) separately. Attempts with H4 (1–15) resulted in a model with poor Z-score and HADDOCK scores. However, attempts with the H4 basic patch (residues 15–21) resulted in a cluster with a Z-score of −1.5 and a HADDOCK score of −62.7+/− 5.7, indicating a favorable interaction. In the resultant model H4 residues 15–21 lie in the narrow groove between the α1 and α2 helices of SANT1 consistent with the NMR data (Fig. 3c). The model predicts that the complex is stabilized at the SANT1 α1 helix by a salt bridge between SANT1 E173 and H4K16, and a hydrogen bond between the backbone carbonyl of SANT1 A177 and the ε-amino group of H4K20. Stabilizing contacts with the SANT1 α2 helix include interactions with H4 H18, which is positioned to form a hydrogen bond with the backbone carbonyl of SANT1 I227 (though the donor-acceptor distance is just over 4 Å in the model) as well as a favorable S/π interaction with M230. Finally, H4 V21 is predicted to be stabilized through van der Waals interactions with SANT1 residues Y181 and I223.

To validate the HADDOCK prediction that SANT1 binds to the basic patch of the H4 tail, two separate H4 peptides (residues 1–10 and 11–21) were tested by NMR. H4(1–10) led to very minor CSPs throughout SANT1, indicative of non-specific interactions similar to that seen for H2A and H2B (Supplementary Fig. S7a). In contrast to this, H4(11–21) led to very similar CSPs as was observed for H4(1–21), indicating that the binding region is within residues 11–21, in agreement with the HADDOCK model (Fig. 3d and Supplementary Fig. S7a). Notably, the computed binding affinity for the shorter H4(11–21) peptide is 0.7 mM +/− 0.2 mM, ~2x weaker than that observed for the H4(1–21) peptide (Supplementary Fig. S7b). This suggests that non-specific interactions with the charged H4 N-terminus are important for binding affinity, but do not contribute to specificity.

### SANT1 provides sensitivity to the modification state of the histone H4 tail

Based on the SANT1/H4 model generated, we would predict that modifications to the basic patch of the H4 tail should modulate the interaction of SANT1. To test this, pull-downs were carried out using biotinylated histone peptides and purified untagged-SRM-SANT1. SRM-SANT1 was incubated with Biotin-tagged H4 peptides unmodified or containing single modifications. Specifically, H4 acetylated at K16 (H4K16ac) or K20 (H4K20ac), or tri-methylated at K20 (H4K20me3) were tested. Reactions were bound to strepdavidin resin, washed extensively, and SANT1 visualized by coomassie stain. Results reveal that acetylation of K16 or methylation of K20 led to significant decreases in SRM-SANT1 binding (Fig. 3e). Acetylation of K20 also decreased binding, though not statistically significantly (Fig. 3e). This is consistent with the HADDOCK model as acetylation of H4K16 would disrupt critical hydrogen bonds/salt bridges, and tri-methylation and acetylation of H4K20 would disrupt the hydrogen bond with the SANT1 backbone and methylation would additionally sterically occlude interaction of Val21 in the hydrophobic pocket. To confirm the pulldown results, an NMR titration of SRM-SANT1 with an H4K16ac peptide was carried out. As seen in Fig. 3f the presence of the acetyl mark substantially abrogates interaction with the H4 tail (also see Supplementary Fig. S7a).

H4K16ac is strongly correlated with active gene transcription whereas H4K20me3 and H4K20ac are correlated with gene repression^35–37^. Analysis of available data sets for H4K20me3, H4K16ac, and H3K27me3 was carried out to determine the extent of overlap of these PTMs. As shown in Fig. 3g there is little overlap between H3K27me3 and H4K20me3 or H4K16ac in IMR90 lung fibroblasts, where data for all three PTMs was available^38–40^. Though there was no deposited data for H4K20ac in the same cell line, a recent report demonstrated that H3K27me3 and H4K20ac are also generally anti-correlated^36^. Together this demonstrates that SANT1 is sensitive to the H4 modification state in a manner consistent with global patterns of PRC2 mediated H3K27me3.

## Discussion

Our results suggest that the SANT1 domain of EZH2 can associate with the H4 N-terminal tail and provide sensitivity to its modification state. The ability of chromatin modification and remodeling complexes to sense the histone modification state of their substrates is critical in their ability to navigate and respond to a complex and dynamic chromatin landscape. This is generally thought to be accomplished through the action of multiple histone reader domains within a given complex, which provide sensitivity to several histone modifications^6^. The PRC2 H3K27 methyltransferase has been shown to be sensitive to the methylation status of H3K27 and H3K4 through the EED and RbAp48 subunits respectively^15–17^. Our results suggest that EZH2 SANT1 provides sensitivity to the modification state of H4K16 and H4K20. Negative regulation of SANT1 binding by the modification of these residues is consistent with the lack of overlap of H4K16ac and H4K20me3 with H3K27me3 in our analysis of IMR90 lung fibroblasts^38–40^. In addition, it is consistent with genome wide functional correlations. Specifically H4K16ac is strongly correlated with active transcription, and it was recently shown that ablation of the H4K16 acetyltransferase MOF in HeLa cells led to a marked increase in H3K27me3^41^. In addition, though H4K20me3 is a repressive PTM, it is correlated with H3K9me3 and generally associated with constitutive heterochromatin, rather than the H3K27me3-rich facultative heterochromatin^35,42^. Additional studies are needed to determine exactly how the SANT1/H4 interaction contributes to PRC2 function.

Several reader domains have been found to specifically associate with H4 carrying various methylation states on Lys20, but only three domains have been found to associate with unmodified H4. The SMRT SANT2 domain was previously shown to associate with the unmodified H4 tail, and was negatively regulated by acetylation, in this case tetra-acetylation of K5, K8, K12 and K16^24^. In addition, cMyb repeat 3 was also found to associate with the H4 tail^26,27^. Comparison of the SMRT SANT2, cMyb repeat 3, and EZH2 SANT1 domains reveal that some residues observed here to be important for complex formation are conserved, suggesting that the SMRT2 SANT2 and cMyb repeat 3 may have a similar mechanism of association with H4. The third domain found to bind to the H4 tail, is the BAH domain of Sir3^43^. Three crystal structures of this domain in complex with the nucleosome revealed an extensive association with both the H4 tail and nucleosome core^44–46^. There is little structural similarity between Sir3 BAH and EZH2 SANT1, however the mode of H4 tail binding observed in the SANT1/H4 model is highly reminiscent of that observed in the Sir3 BAH/NCP structure. The BAH domain contacts the H4 tail via hydrogen bonds and van der Waals contacts with H4 residues K16, H18, K20, V(I)21 and K23, while R17 and R19 face away from the BAH domain and make contacts with the nucleosomal DNA. This mechanism of recognition and the conformation of the histone tail is almost identical to what is seen in the SANT1/H4 model.

A cryo-EM structure of PRC2 in complex with a di-nucleosome was recently solved^47^. This structure along with previous biochemical analyses reveals that the affinity of the complex is dominated by robust interactions with DNA, whereas regulation and potentially orientation of the complex are determined by weaker associations with the histone tails^48,49^. This is consistent with the weak binding affinity seen for SANT1/H4. Notably, in the crystal structures of the PRC2 complex without nucleosomes SANT1 is seen to associate with a region of EZH2 termed the SANT binding domain (SBD, see Fig. 1a). However, in the PRC2/di-nucleosome structure, the SBD undergoes a conformational change and the SANT1 domain is seen to be highly flexible, suggesting that it is available to associate with H4. SANT1 does not make any noticeable contacts with the di-nucleosome, however, it is perfectly positioned to make contacts with an adjacent H4 tail in a tetranucleosome configuration^50,51^. PRC2 is the most active toward compacted chromatin arrays, thus it is possible that the SANT1 domain not only helps to position the complex at this substrate, but to provide a sensor for the modification state of the H4 tail. It is tempting to suggest that this could be communicated to the SRM through the SRM-SANT1 linkage, but this would require further extensive studies.

## Methods

### Cloning, expression and purification of EZH2 SANT1

The complete human EZH2 cDNA was obtained from OpenBiosystems (GE Healthcare, Accession #:BC010858). EZH2 SANT1 (residues 159–251) and SRM-SANT1 (residues 141–251) were subcloned into a pDEST15 (N-terminal GST-tag) expression vector using the Gateway Recombination cloning method (Invitrogen, ThermoScientific) with a PreScission Protease cleavage site engineered between the GST-tag and the N-terminus of EZH2 SRM-SANT1. Mutants were generated in the 141–251 construct using site-directed mutagenesis (Q5 mutagenesis kit, New England Biolabs).

All EZH2 constructs were expressed in *E*. *coli* Rosetta2 (DE3) pLysS cells that were grown in LB medium or M9 minimal media supplemented with 1 g/L ^15^NH_4_Cl and 5 g/L D-glucose (for uniformly ^15^N-isotopically enriched protein) or 3 g/L ^13^C-D-glucose (for uniformly ^15^N/^13^C-isotopically enriched protein). For expression in LB medium, cells were grown to an OD_600_ ~ 1.0 and induced with 1.0 mM IPTG for 16 hours at 20 °C. For expression in M9 minimal media, cells were grown in 3–4 L LB medium until and OD_600_ ~ 1.5 then spun down for 15 minutes at 4000 g and re-suspended in 1 L M9 minimal media^52^. After transfer, cells were allowed to equilibrate shaking at 20 °C for one hour before induction with 1.0 mM IPTG for 18 hours at 20 °C. Cells were collected by centrifugation for 20 minutes at 4000 g the pellet was flash frozen in liquid N_2_ and stored at −80 °C.

For purification, cell pellets were thawed on ice and lysed by emulsiflex in a lysis buffer containing 150 mM NaCl, 50 mM Tris (pH 6.5), 3 mM DTT, 0.1% Triton X-100 and DNaseI. Lysate was cleared at 18,000 g for 1 hour at 4 °C. The soluble supernatant was incubated with glutathione agarose resin (ThermoFisher Scientific) and washed extensively with a buffer containing 900 mM NaCl, 50 mM Tris (pH 6.5) and 3 mM DTT. The GST-tag was cleaved by incubation with PreScission Protease overnight and SRM-SANT1 was further purified by FPLC using anion exchange over a Source15S column and size exclusion chromatography over a superdex 75 column. The final buffer contained 150 mM NaCl, 50 mM Tris (pH 6.5), 3 mM DTT, with or without 5 mM EDTA.

### Resonance assignments and Chemical Shift Indexing

To determine backbone resonance assignments, HNCACB, HN(CO)CACB, CBCA(CO)NH, HNCO, HNCACO and HCCH-TOCSY experiments were collected on ^15^N/^13^C-EZH2 SRM-SANT1 at a concentration of 0.7 mM on a Unity Inova 600 MHz Oxford AS600 spectrometer or Bruker AVANCE II 500 MHz spectrometer with a 5 mm triple resonance probe at 25 °C. All data was processed in NMRpipe and further analyzed using CcpNmr^53,54^. Initial assignments were generated using the PINE server and further curated manually using CcpNmr^54,55^.

Secondary structure propensity prediction was carried out using the program TALOS+ ^33^. The HN, N, Cα, Cβ and CO chemical shifts for all EZH2 SANT1 residues that were unambiguously assigned were provided as input.

### Electrophoretic Mobility Shift Assays (EMSAs)

601 DNA was purified as previously described^56^. Samples were prepared by mixing 1.5 pmol of DNA with varying amounts of EZH2 SRM-SANT1 to reach final molar ratios of DNA:SRM-SANT1 of 1:0, 1:1, 1:5, 1:10, 1:15, 1:20 and 1:50 in a final buffer containing 12.5 mM NaCl, 25 mM Tris (pH 6.5), 1.5 mM DTT, and 5% v/v sucrose for purposes of gel loading. Samples were allowed to equilibrate for 1 hour on ice before loading onto 5% native 59:1polyacrylamide gel, run with 0.2x TBE. Native gels were run on ice in a 4 °C cold room for 45 minutes at 125 V, stained with ethidium bromide and visualized using an ImageQuant LAS 4000 imager. SRM-SANT1 EMSAs with 601 DNA were performed in duplicate.

### NMR titrations and calculation of dissociation constants

Unmodified histone H2A (1–21, AS-63676), histone H2B (1–11, 65442-1), histone H3 (1–21, AS-61701 and 21–44, AS-64454-1) and histone H4 (1–21, AS-62499) peptides were obtained from AnaSpec. Unmodified histone H4 (1–10 and 11–21) were custom synthesized by GenScript. Biotinylated histone H4K16ac (1–21, AS-64847-1) was obtained from Anaspec. All histone peptides were dissolved in H_2_O at a concentration of 20 mM, and the pH adjusted to ~7.0. DNA oligos were obtained from IDT. The DNA was annealed by mixing equimolar amounts of single stranded oligos. DNA was heated to 95 °C for 10 minutes before allowing to cool overnight. The annealed DNA was further purified by size exclusion chromatography over a superdex 75 column in a buffer containing 150 mM NaCl, 50 mM Tris (pH 6.5), 3 mM DTT, with 5 mM EDTA. Titrations were performed by collecting ^15^N-HSQC spectra on 0.05–0.10 mM ^15^N-EZH2 SRM-SANT1 while titrating in increasing concentrations of the respective ligands. Titration experiments were performed at 25 °C on a Bruker Avance II 800 MHz NMR spectrometer equipped with a 5 mm triple resonance cryoprobe. Data was processed and analyzed using NMRPipe and CcpNmr^53,54^. Dissociation constants (K_d_) were calculated by nonlinear least-squares analysis in GraphPad PRISM to a one-site binding model accounting for ligand depletion using the equation:

1 $${\rm{\Delta }}\delta ={\rm{\Delta }}{\delta }_{max}(([L]+[P]+{K}_{d})-\sqrt{{([L]+[P]+{K}_{d})}^{2}-4[P][L]})/(2[P])$$

where [L] is the concentration of histone peptide, [P] is the concentration of the protein, Δ*δ* is the observed normalized chemical shift change and Δ*δ*_max_ is the normalized chemical shift change at saturation, calculated as

2 $${\rm{\Delta }}\delta =\sqrt{{({\rm{\Delta }}{\delta }_{H})}^{2}+{(0.20{\rm{\Delta }}{\delta }_{N})}^{2}}$$

where Δδ is the chemical shift in ppm.

K_d_ values were determined for all individual residues with significant Δδ in each individual titration. A global K_d_ for each peptide was determined by the averaging the K_d_ of all individual residues that were significantly perturbed in the titration. For this analysis, a Δδ value was considered significant if it was greater than the average Δδ + 1.00 standard deviations of all residues (excluding unassigned residues) after trimming the 10% of residues with the largest Δδ. Individual residues with a K_d_ value greater than or equal to 2 standard deviations from the global mean K_d_ for that specific histone peptide titration were removed from the analysis. If 50% or more of the individual residue K_d_ values were greater than 2 standard deviations from the global mean K_d_, than the global K_d_ is reported as greater than the K_d_ of the highest affinity individual residue (i.e. >2.0 mM).

### Scanning Wavelength Circular Dichroism

Scanning wavelength CD spectra were obtained on 25 μM samples of EZH2 SRM-SANT1 proteins in a buffer containing 20 mM sodium phosphate (pH 6.4) at 25 °C using a Jasco J-815 CD spectrometer. Three scans were collected for each spectra scanning from 195–260 nm, and subtracted from a single spectrum containing buffer only.

### HADDOCK Modeling

Modeling of the EZH2 SANT1:H4 tail complex was performed using HADDOCK^57^. Input coordinates for EZH2 SANT1 (residues 160–241) and the histone H4 tail were taken from 5HYN and 1KX5, respectively. Residues V172, E173, A177, Q180, Y181, D221, K222, I223, E225, A226, M230, T236 and E242 of EZH2 SANT1 and residues A15, K16, R17, H18, R19, K20, and V21 of the histone H4 tail were defined as active residues in assigning unambiguous restraints. Residues H4 (15–21) were chosen based on better scoring clusters than models generated with H4 (residues 1–15) Modeling was carried out on the HADDOCK2.2 webserver^34^ and results analyzed using PyMOL^58^. The highest scoring model from the top scoring cluster of models was used.

### Biotinylated histone peptide pulldown

Biotinylated unmodified histone H4 (1–23, SKU:12-0029), unmodified H4 (11–27, SKU:12-0035), H4K16ac (1–23, SKU:12-0033), H4K20ac (1–24, SKU:12-0052) and H4K20me3 (11–27, SKU:12-0038) peptides were obtained from EpiCypher. 1 μg of purified SRM-SANT1 was incubated with 1 μg of Biotin-tagged H4 peptides unmodified or containing single modifications. Reactions were incubated overnight rocking end over end at 4 °C in a binding buffer containing 150 mM NaCl, 50 mM Tris (pH 6.5), 3 mM DTT. Streptavidin sepharose resin (25 μL of 50% slurry, GE Life Sciences) was added to each reaction and rocked end over end at 4 °C for two hours. The reactions were then spun at 2500 × g, supernatant decanted and beads washed with 500 μL of the binding buffer four times. After the final wash, beads were resuspended in 5x SDS loading dye, ran on a 4–20% gradient gel and visualized using coomassie staining. Gels were imaged using an ImageQuant LAS 4000 imager. Quantification of triplicate pull down experiments was performed using ImageJ^59^.

### ChIP-seq peak overlap

BED files for H3K27me3, H4K16ac, and H4K20me3 in IMR90 lung fibroblasts were downloaded from NCBI Gene Expression Omnibus (GEO) database (Accessions: GSE38442, GSE56307, GSE59316, respectively). Peaks for H3K27me3 were called in reference to hg18 while H4K20me3 and H4K16ac were called to hg19. Galaxy was used to convert GSE38442 to hg19 coordinates for further analysis. All data sets were imported into R Studio. Peak overlaps were determined using the ChIPpeakAnno package^60,61^. Overlaps were defined as being within 150 base pairs of each other and on the same strand.

## Supplementary information

Supplementary Figures

## Data Availability

The NMR assignments have been deposited to the Biological Magnetic Resonance Data Bank (BRMB) with accession number 27706. The authors declare that data supporting the findings of this study are available within the paper and its supplementary information files, raw data and plasmids are available from the corresponding author upon reasonable request.
