# Supplementary material for: The EZH2 SANT1 domain is a histone reader providing sensitivity to the modification state of the H4 tail
Source: Sci Rep. 2019 Jan 30;9:987. doi: 10.1038/s41598-018-37699-w (PMC6353875; doi:10.1038/s41598-018-37699-w)
Supplement: Supplementary file 1 — Supplementary Figures [file 41598_2018_37699_MOESM1_ESM.pdf]

The EZH2 SANT1 domain is a histone reader providing sensitivity to the modification state of the H4 tail

Tyler M. Weaver[1], Jiachen Liu[1], Katelyn E. Connelly[2], Chris Coble[1], Katayoun Varzavand[1], Emily C. Dykhuizen[2], Catherine A. Musselman[1]*

Supplementary Data
(Figures S1-S9)

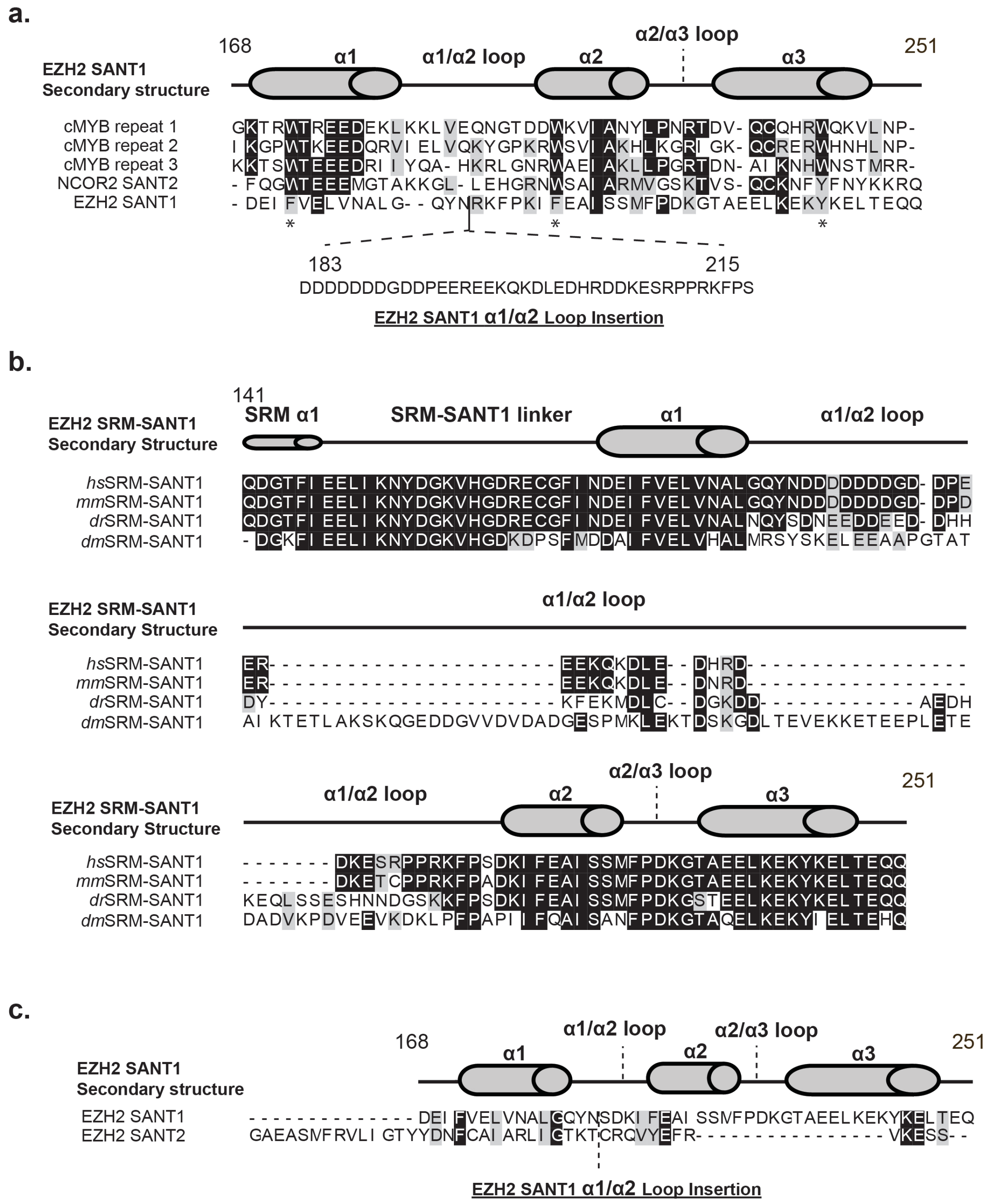

Supplementary Figure 1. Sequence Alignments. (a) Multiple sequence alignment of selected SANT/Myb domains show conservation of core residues. Residues that are part of the SANT1 hydrophobic core are denoted with *. An extended α1/α2 loop found in EZH2 is shown below the alignment of the core SANT/Myb structured region. **(b)** Alignment of SANT1 sequences across several species show that the core domain is well-conserved across higher eukaryotes. **(c)** Sequence alignments of EZH2 SANT1 and EZH2 SANT2 show little sequence conservation between the domains. Sequence alignments were generated using Clustal Omega and the BoxShade server. Residues with >0.5 sequence identity are highlighted black and non-identical residues that contain similar amino acid types are highlighted grey. The TALOS+ predicted secondary structure of SRM-SANT1 is represented above the sequence alignments.

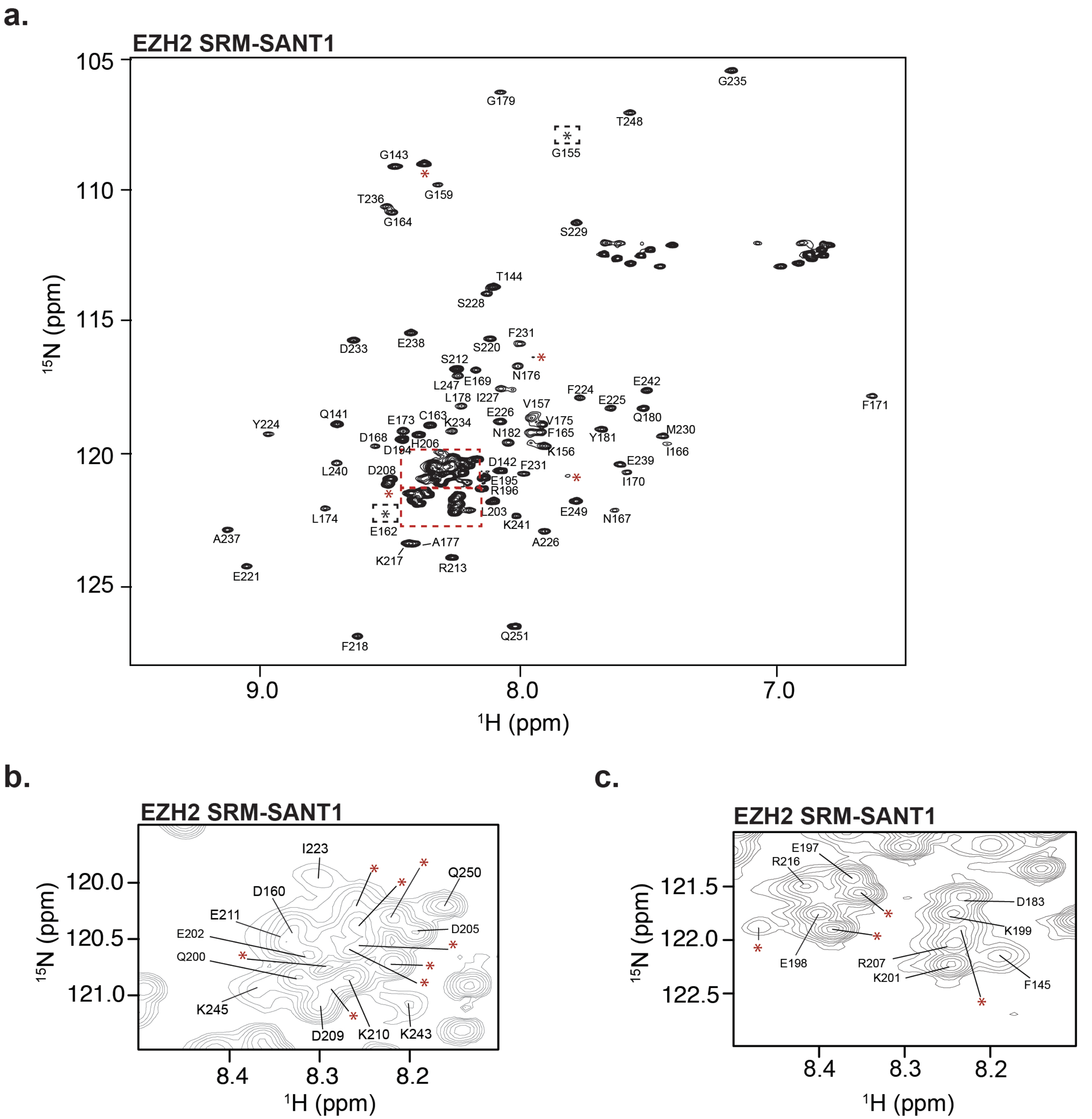

Supplementary Figure 2. SRM-SANT1 Assignments. (a) ^{1}H-^{15}N HSQC spectrum of the SRM-SANT1 domain used for assignments. Assigned resonances are labeled with one-letter amino acid nomenclature. Unassigned peaks are denoted with a red asterisk. Low intensity peaks are boxed in black and labeled with a black asterisk. The regions outlined by red dashed boxes are shown expanded in **(b) and (c).**

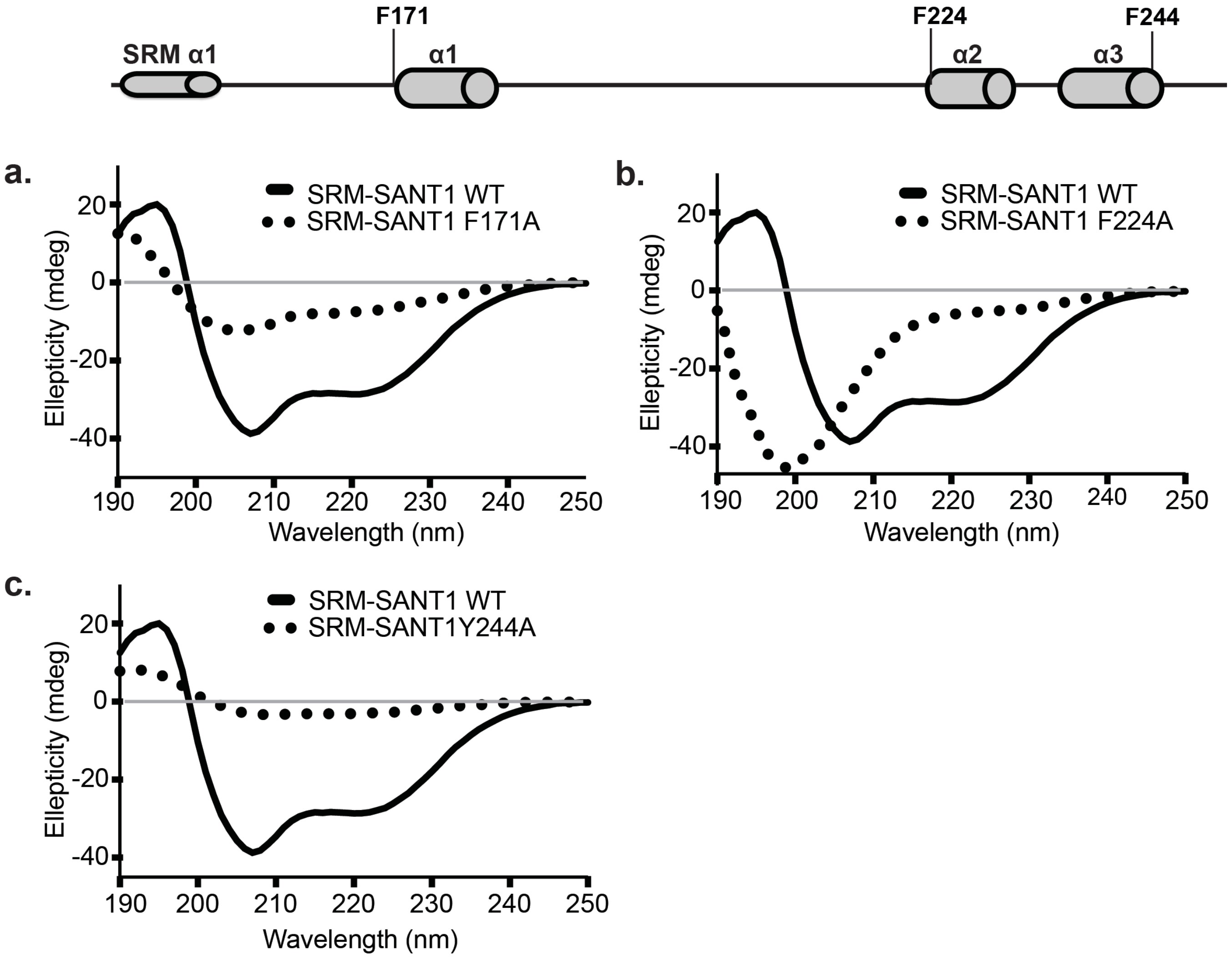

Supplementary Figure 3. Structural characterization of EZH2 SRM-SANT1. Overlays of scanning wavelength circular dichroism (CD) spectra of SRM-SANT1 wild-type (WT, black solid line) with **(a)** F171A (black dots), **(b)** F224A (black dots), **(c)** Y244A (black dots).

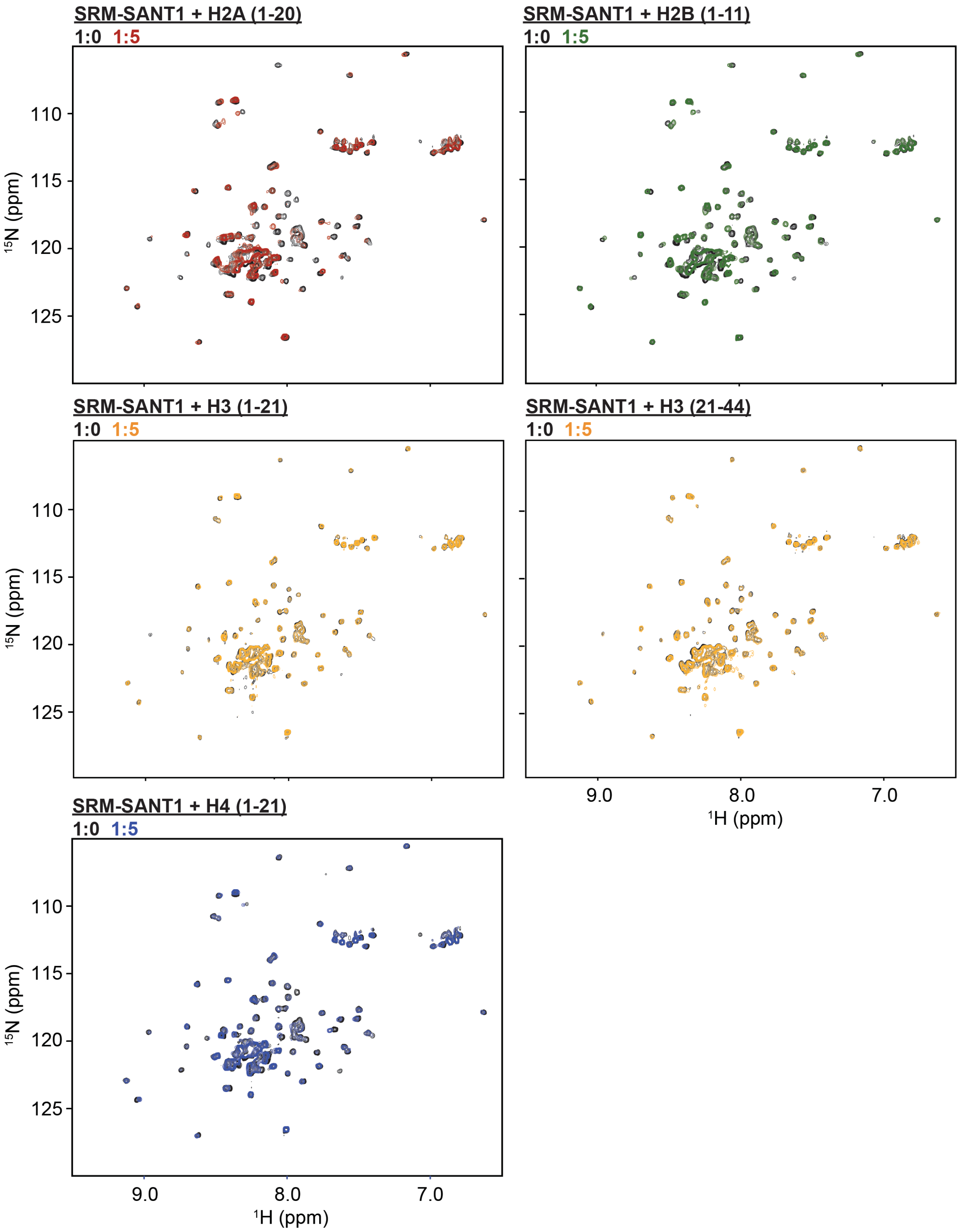

Supplementary Figure 4. Full Histone Titrations. Full ^{1}H-^{15}N HSQC overlays of the SRM-SANT1 titrations with all unmodified histone peptides. All apo-state spectra are colored black. Spectra of the highest SANT1:peptide ratio (1:5) are colored red for H2A (1-20), green for H2B (1-11), orange for H3 (1-21 and 21-44) and blue for H4 (1-21).

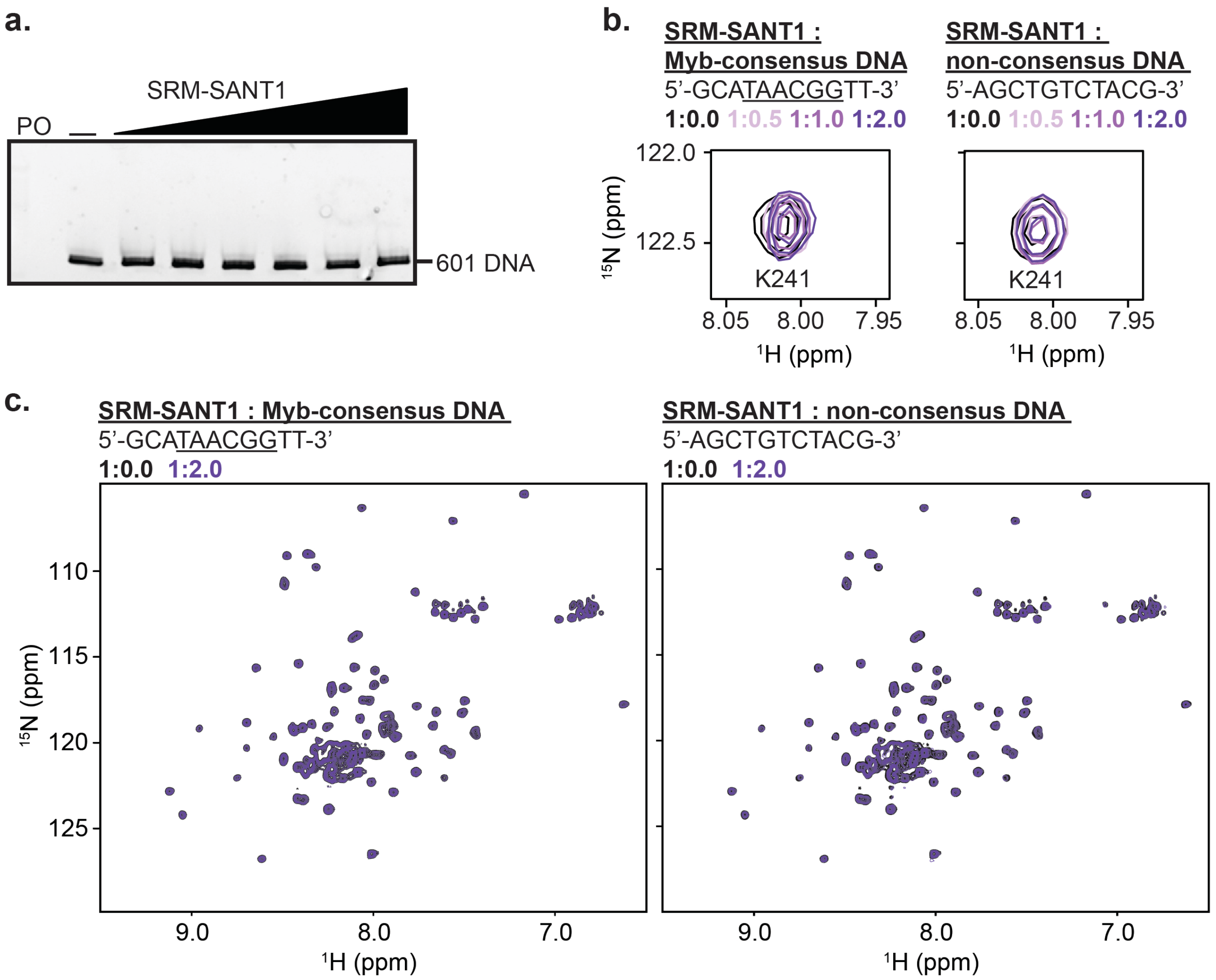

Figure 5. SRM-SANT1 is not a DNA binding domain. (a) Electromobility shift assay (EMSA) of the 147bp Widom 601 DNA sequence in the presence of increasing concentration of SRM-SANT1. PO denotes protein only and '–' denotes 601 DNA only. ^{1}H-^{15}N HSQC overlay for α3 residues K241 in the presence of increasing concentrations of **(b)** an 11bp DNA containing the Myb-consensus sequence (underlined) or **(c)** an arbitrary 11bp DNA sequence. Molar ratios of SRM-SANT1:DNA are shown in the legend above the overlays. **(d)** Corresponding full ^{1}H-^{15}N HSQC overlays of the SRM-SANT1 titrations with 11bp Myb-consensus DNA sequence (left) or arbitrary DNA sequence (right). The apo-state spectra are colored black and spectra at the highest ratio of SANT1:DNA (1:2) colored purple.

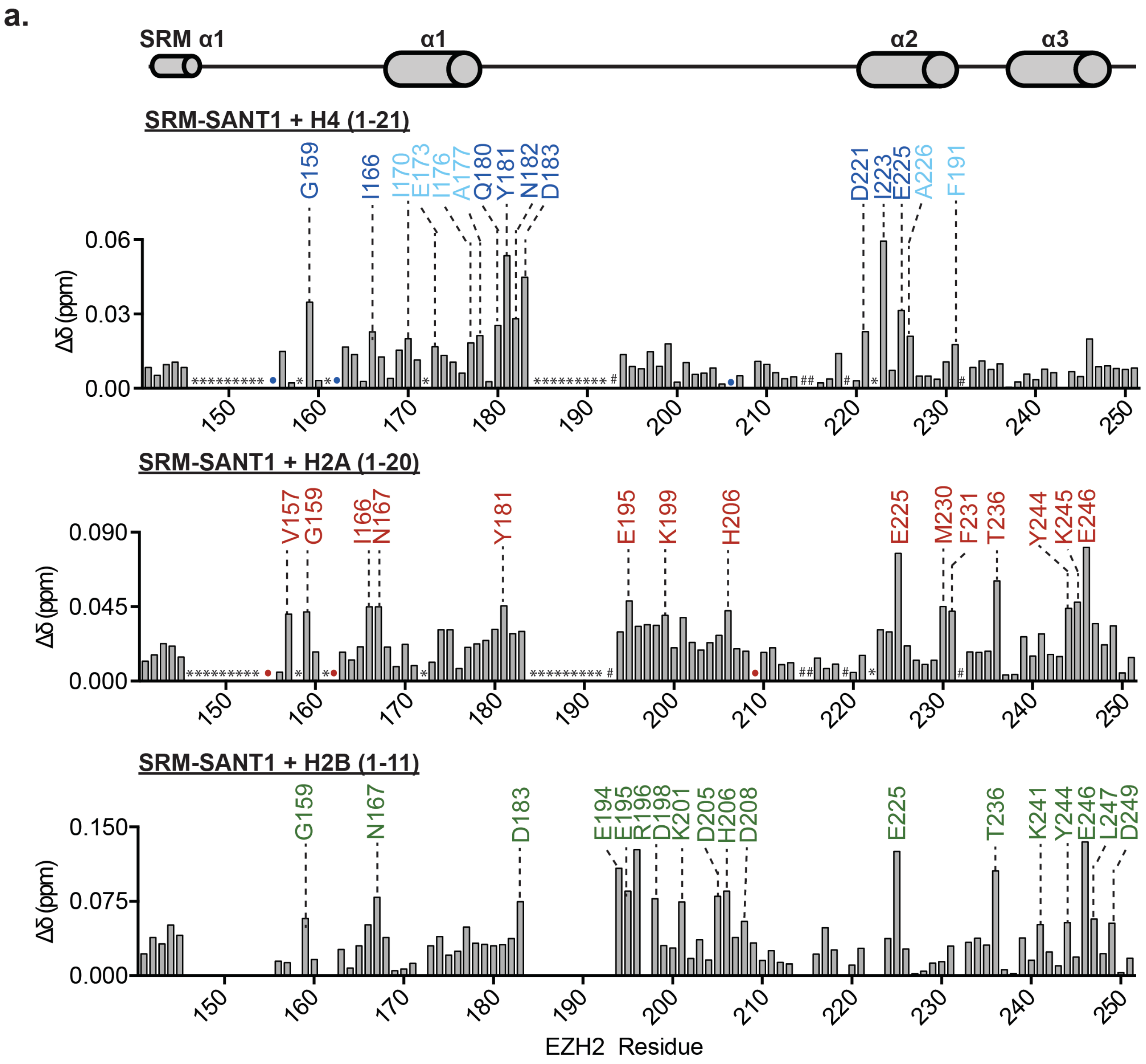

Supplementary Figure 6. H2A and H2B bind non-specifically. Normalized chemical shift perturbation (Δδ) in the ^{1}H-^{15}N HSQC of ^{15}N-SRM-SANT1 between apo and H4-bound (top), H2A-bound (middle), or H2B-bound (bottom) as a function of EZH2 residue. TALOS+ predicted secondary structure is shown above. H4 Residues with significant chemical shift perturbation greater than 1.0 and 1.5 standard deviations from the mean are labeled with cyan and blue, respectively. H2A and H2B residues with significant chemical shift perturbation greater than the mean plus 1.0 standard deviation are labeled in red and green, respectively. Unassigned residues are indicated with *. Prolines are denoted with #. Colored dots represent resonances that broaden beyond detection in the titration experiment.

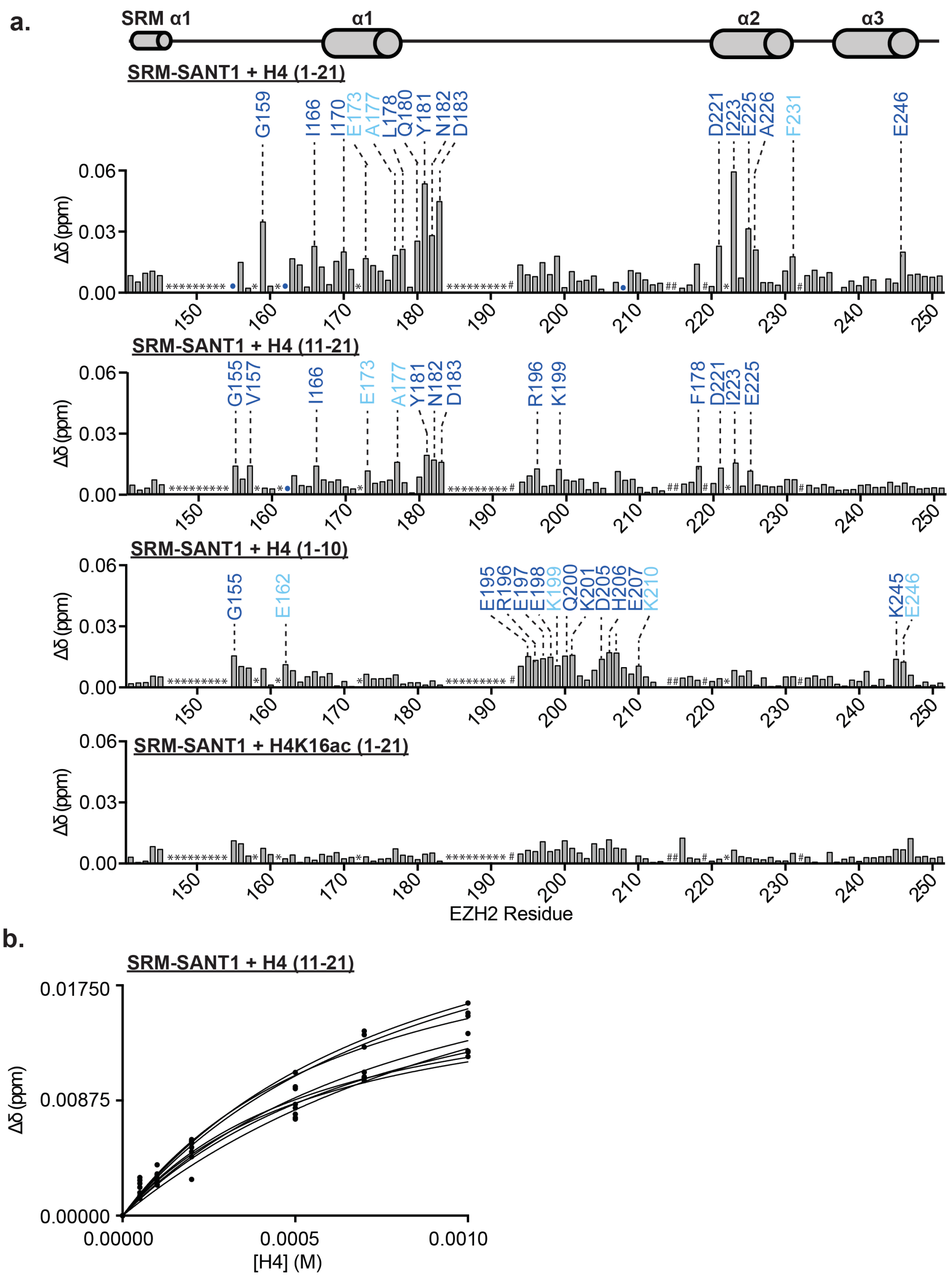

Supplementary Figure 7. SRM-SANT1 binds specifically to the H4 basic patch. (a) Normalized chemical shift perturbations (Δδ) in the ^{1}H-^{15}N HSQC of ^{15}N-SRM-SANT1 between apo and when bound to H4(1-21), H4(11-21), H4(1-10), or H4K16ac(1-21) as a function of EZH2 residue. TALOS+ predicted secondary structure is shown above. Residues with significant chemical shift perturbation greater than the mean plus 1.0 and 1.5 standard deviations are labeled with cyan and blue, respectively. Unassigned residues are indicated with *. Prolines are denoted with #. Colored dots represent resonances that broaden beyond detection in the titration experiment **(b)** Binding curves used to calculate the K_d of SRM-SANT1 for H4(11-21).

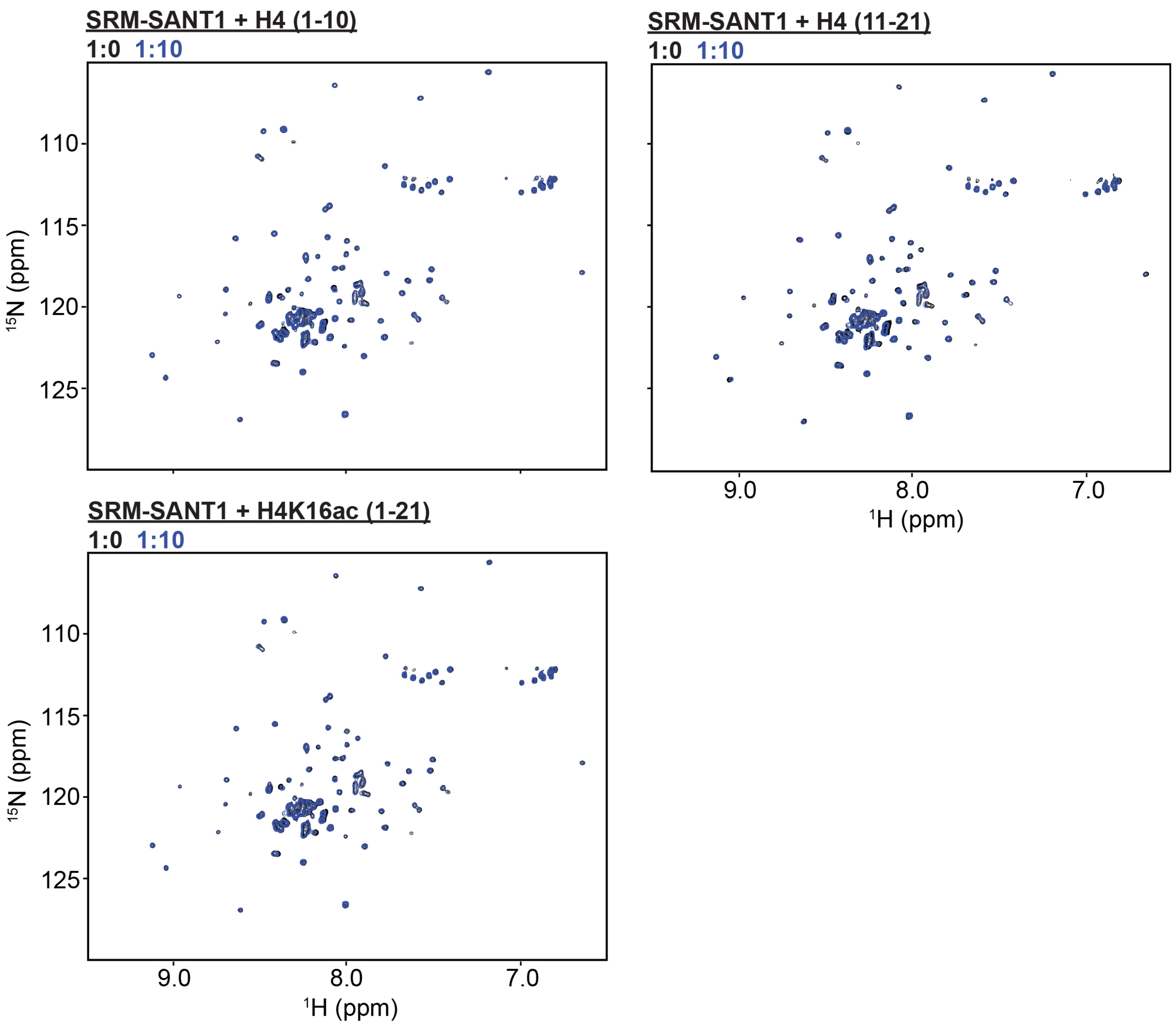

Supplementary Figure 8. Full H4 Peptide Titrations. Full ^{1}H-^{15}N HSQC overlays of the SRM-SANT1 titrations with all H4 histone peptides. All apo-state spectra are colored black. Spectra of the highest SANT1:peptide ratio (1:10) are colored blue and shown for H4(1-10), H4(11-21), and H4K16ac(1-21).

a.

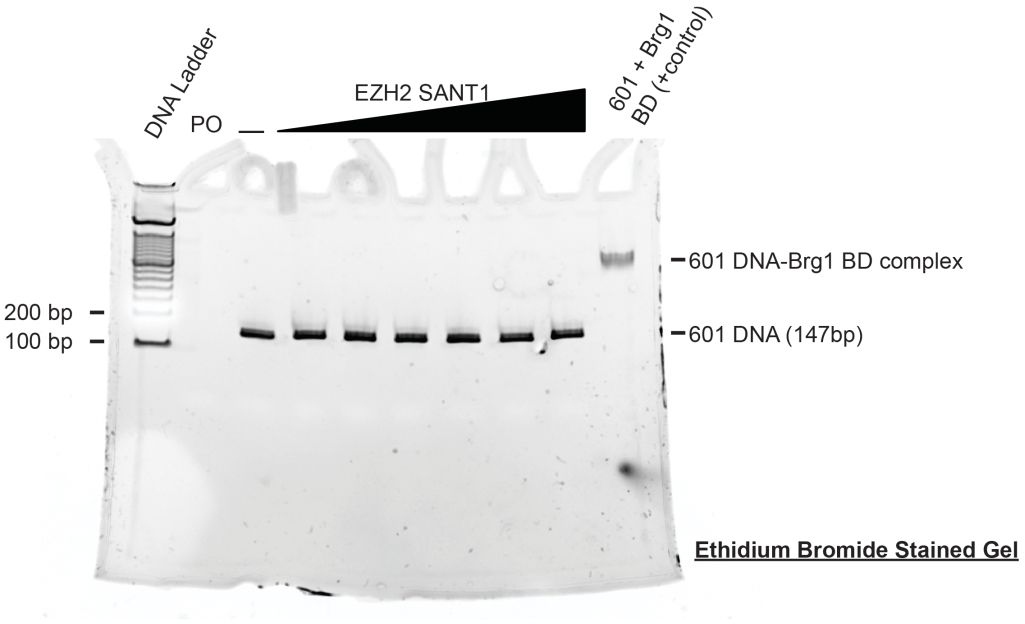

b.

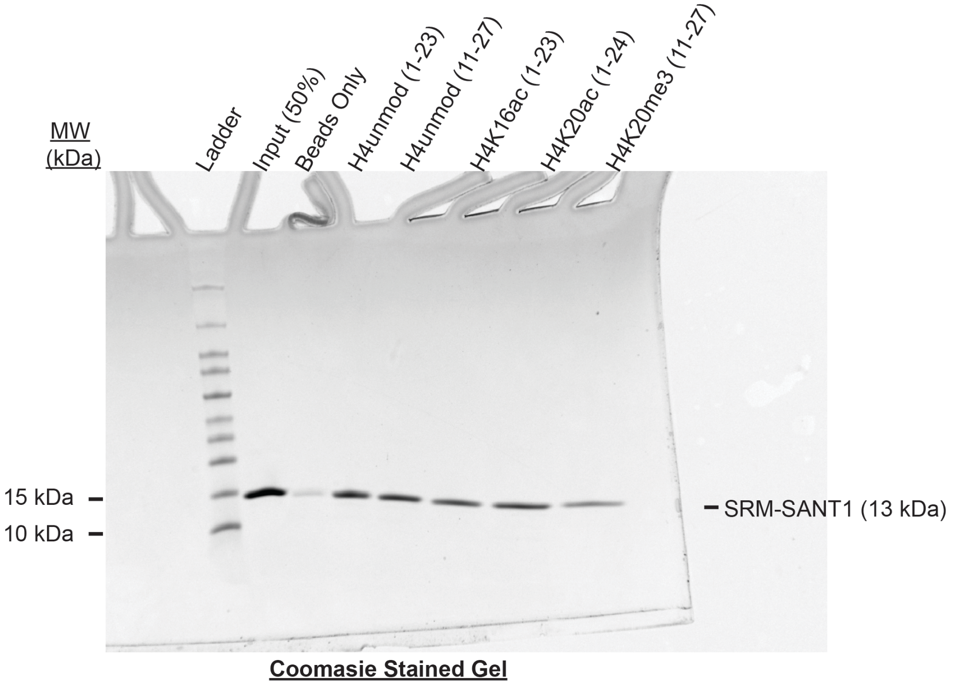

Supplementary Figure 9. Full gel images. (a) A representative full gel image of the EMSA of SRM-SANT1 with 601 DNA (see corresponding Fig. 3). Shown are a 100bp ladder, protein only (PO), 601 DNA only (—), and 601 DNA in the presence of increasing concentrations of EZH2 SRM-SANT1. A positive control of the BRG1 BD known to bind DNA is shown on the far right. **(b)** A representative full gel of the SRM-SANT1 pulldown with biotinylated modified histone peptides (see corresponding Fig. 7).
